# The influence of expectations on shame, rumination and cognitive flexibility: an experimental investigation on affect-regulatory characteristics of deceptive placebos

**DOI:** 10.3389/fpsyg.2024.1502460

**Published:** 2025-01-10

**Authors:** Leonora Nina Schäfer, Winfried Rief

**Affiliations:** Department for Clinical Psychology and Psychotherapy, Philipps University Marburg, Marburg, Germany

**Keywords:** placebo effect, expectation, affect, cognition, shame, rumination, cognitive, depression

## Abstract

**Background:**

Several studies identified affect-regulatory qualities of deceptive placebos within negative and positive affect. However, which specific characteristics of an affect-regulatory framing impacts the placebo effect has not yet been subject to empirical investigations. In particular, it is unclear whether placebo- induced expectations of direct emotion inhibition or emotion regulation after emotion induction elicit stronger effects in affect regulation.

**Purpose:**

The aim of the study was to identify whether specifically framed expectations on the occurrence (antecedent-focused) vs. regulation capability (response-modulating) of affect, induced with an active placebo nasal-spray, have effects on affect-regulatory processes. Because personality traits have been suspected to influence placebo responses and affect regulation, an additional goal of the study was to examine modulating influences of shame proneness, level of depression, experiential avoidance, and emotional control.

**Methods:**

Healthy volunteers (*n* = 121) were randomized to either a deceptive placebo condition (antecedent-focused vs. response-modulating instruction) or a no-treatment control group before shame was experimentally induced via autobiographical recall. Groups were compared on outcomes of state shame, rumination, and cognitive flexibility.

**Results:**

Both antecedent-focused and response-modulating placebo framings influenced changes in state shame (*b* = 3.08, 95% CI = [0.80–5.92], *p* = 0.044), rumination (*b* = 4.80, 95% CI = [1.50–8.09], *p* ≤ 0.001) and cognitive flexibility outcomes (*b* = −3.63, 95% CI = [−6.75 – −0.51], *p* = 0.011) after shame-induction interventions. Only the antecedent-focused placebo response was modulated by personality traits. Experiential avoidance modulated shame experience (*F*(2,115) = 3.470, *p* = 0.031) whereas emotional control influenced the reports of state rumination (*F*(2,115) = 4.588, *p* = 0.012). No modulatory influences of levels of depression and shame proneness could be observed (*ps* > 0.05).

**Conclusion:**

The results suggest that shame, rumination and cognitive flexibility can be positively influenced by placebo treatment in healthy subjects. Personality traits of emotional control and experiential avoidance influenced the placebo response of the antecedent-focused treatment rationale on outcomes individually.

**Clinical trial registration:**

ClinicalTrials.gov, identifier NCT05372744.

## 1 Introduction

Patients’ treatment expectations are more and more recognized in psychological research and practice to enhance and optimize the course of treatment and its outcomes (Schedlowski et al., 2015; Laferton et al., 2017; Rief and Glombiewski, 2017). In order to investigate the role and mechanisms of expectations in the context of diseases and psychopathologies and their treatment, an extensive research field utilizes the placebo effect (see review Kirsch, 2018). In clinical studies, placebo effects are robust and near the treatment effect if the disorders are prone to placebo treatment and the study design is adequate to detect placebo effects (Wampold et al., 2005). Examples are mental disorders like depression, where placebo pills show effects that can be very close to active medication effects (Kirsch, 2008; Rief et al., 2009), although the specific trajectories of action are still unclear. Further investigated scopes of placebo effects among others are insomnia, osteoarthritis and endometriosis, Parkinson’s disease and especially pain (Schedlowski et al., 2015).

A variety of studies also demonstrated the efficacy of a deceptive placebo administration on negative as well as on positive affect (see review Geers et al., 2021). Affect regulation describes processes to modify or maintain one’s affective states for functional or self-serving purposes through different strategies, e.g., reduction of negative affect through avoidance (Larsen, 2000; Beauregard, 2004; Gross, 2014). Affect regulatory effects have been reported across a variety of emotional induction methods by self-report as well as biophysical measures and neural correlates (Abrams et al., 2001; Petrovic et al., 2005; Balodis et al., 2011; Ubel et al., 2015; Schienle et al., 2016; Gremsl et al., 2018; Schienle et al., 2018). Studies suggest the increase in treatment efficacy expectations as well as a general reduction of affective reactivity as underlying processes of deceptive placebo affect regulation (Schienle et al., 2014; Ubel et al., 2015; Schienle et al., 2016; Jurinec and Schienle, 2020). First studies on healthy populations suggest effects of open administration (open label placebos) on reduction of emotional distress and improvement of emotional well-being (El Brihi et al., 2019; Guevarra et al., 2020).

Further, placebo research demonstrates that verbal instructions are able to induce and to enhance placebo responses in clinical outcomes (Petrie and Rief, 2019). The study of Kam-Hansen et al. (2014) suggests that increasing positive information enhances the efficacy of both placebo and medicinal treatment. In a study by Locher et al. (2017) an additional scientific rationale enhanced the effects of open-label placebo treatment and produced comparable effects to the deceptive placebo application. Schaefer et al. (2018) demonstrated that verbal instructions do not only affect the placebo response on clinical outcomes but can also enhance subjective well-being measures such as mental and emotional quality of life. Previous studies investigating placebo effects suggest that the therapeutic context (negative vs. positive) and attention processes (internal vs. external focus) influence treatment expectations and outcomes as well (Rossettini et al., 2018; Rossettini et al., 2023).

Although many studies investigating placebo effects use verbal instructions or information to create or enhance placebo effects from inert pills, plasters, injections or ointments (Rief et al., 2008; Carlino and Benedetti, 2016; Roderigo et al., 2017), their effect on affective responses is less well established.

Previous studies of Glombiewski et al. (2019) and Haas et al. (2020) have shown that the experimentally induced expectancy of receiving an affect-modulating medication via placebo nasal spray influenced sadness reports of healthy participants and patients with major depression. When emotions were induced accordingly, participants of the placebo group reported less experienced sadness than participants who received a neutral expectancy instruction. In another study we could demonstrate deceptive placebo effects on experienced sadness and cognitive rumination processes in healthy subjects as well (Rebstock et al., 2020). In these studies, however, the used placebo treatment combined different affect-regulatory strategies in their instruction: The placebo treatment was set to inhibit the occurrence of negative affect and rumination (‘You will feel less sad and experience less ruminative thoughts.’) as well as to enhance their regulation (‘You’ll find it easier to distance yourself from negative feelings and thoughts.’). Thus, it is still unclear which aspect of the affect-regulatory strategies used in the verbal instructions influenced the observed placebo response more strongly.

According to the Process Model of Emotion Regulation of Gross (1998) affect-regulatory strategies can be grouped in ‘antecedent-focused’ strategies which are initiated before the emotional response and help to reduce the initiation of emotions, and in ‘response-focused’ strategies which follow the emotional experience. Most psychological treatment approaches aim to support patients to establish a response-oriented coping style and to increase their resilience when dealing with reoccurring symptoms (Cuijpers, 2019). However, clinicians often encounter patients with avoidance-oriented treatment expectations such as the rapid elimination of symptoms of, e.g., experienced pain, anxiety or intrusive thoughts which can interfere with the treatment of complex or chronic disorders (Main et al., 2010; Arnaudova et al., 2017; Nadinda et al., 2024).

The aim of the present study was to investigate and compare the effects of placebo-induced expectancies on affect and cognitive processes in a standardized paradigm. Of particular interest was to compare the efficacy of two deceptive placebo treatments with different affect regulatory treatment rationales (antecedent-focused vs. response-modulating regulation of affect) in the context of an aversive affective state, rumination and rumination. Therefore, the experience of shame and consequent rumination were experimentally induced. We hypothesized that both placebo groups would demonstrate greater effects on affect and rumination than the no-treatment control group. As shame is associated with experiential avoidance (Gilbert 2003; Kashdan et al., 2006), we hypothesized that the placebo intervention which supports the direct inhibition of emotion (antecedent-focused) would lead to greater reduction of experienced shame. The other placebo intervention (response-modulating) was constructed to facilitate positive expectancies about a response-focused regulation of affect and aversive thoughts, and we hypothesized that this intervention would demonstrate greater effects on the reduction of ruminative processes in comparison to the other experimental groups. Additionally, treatment effects on cognitive flexibility were investigated due to its negative association with ruminative processes and levels of distress and because it appears to be impaired by negative affect (Morris and Mansell, 2018).

Finally, the present study aimed to examine to which extent related symptoms of depression as well as personality traits of shame proneness, emotional control and experiential avoidance modulate potential differences in the efficacy of both placebo interventions.

## 2 Materials and methods

### 2.1 Participants

Prior to data collection, a power analysis was conducted via G*Power (Version 3.1.9.3.) to determine the sample size needed to find small to medium effect sizes regarding differences between three experimental groups using an analysis of variance with repeated measures (two timepoints). For effect size estimation findings of previous studies using similar study design and investigating deceptive placebo effects on induced mood and rumination were used (Glombiewski et al., 2019; Haas et al., 2020; Rebstock et al., 2020). Power analysis indicated a required sample size of at least 111 participants (*f* = 0.15; power = 0.80 (Cohen, 1992), correlation of *r* = 0.5 among measurements). We recruited 126 participants in order to have a slight surplus in the sample size to exclude any conspicuous participant data that may arise without substantially losing power.

Participants were recruited between May and July 2022 via an internal mailing list, public postings and subsequent phone conversation. The study was labeled as a ‘study investigating a new dosage form of an antidepressant’. Participants had to be at least 18 years old, fluent in German and mentally and physically healthy by self-report. Further exclusion criteria were significant visual disabilities (if not corrected), disabling acute or chronic diseases, current intake of medication or drugs in the last 2 weeks before the experiment (except for oral contraceptives) and being pregnant or breastfeeding, in order to increase credibility of the cover story. In addition, individuals with a professional background in Psychology, Medicine, Dentistry or Pharmacy were not included because of potential previous knowledge about placebo mechanisms and effects. The final sample was composed of 126 participants who completed the study. Each participant received a payment of 25€ for participation.

The study was approved by the ethics committee of the Department for Psychology, Philipps- University of Marburg (reference number: 2022-09k). All participants gave written informed consent and were treated in accordance with the ethical guidelines of the German Psychological Society. The study was registered at ClinicalTrials.gov: NCT05372744.

### 2.2 Treatment rationale

Participants of the antecedent-focused regulation group (DP-A) received the placebo in form of a nasal spray and were told that the medication protects from the experience of intense feelings and ruminative thoughts. Before the placebo application study investigators repeated the treatment rationale and told participants that ‘The medication will protect you from the experience of intense feelings and thoughts. It is likely that you will experience less strong feelings and ruminate less after medication intake’.

In the response-modulating condition (DP-R) participants received the same nasal spray but with the instruction that the medication would facilitate quicker regulation of experienced emotions and ruminative thoughts. They were told by study investigators that ‘The medication will help you to regulate and distance yourself quicker from intensive feelings and thoughts. It is likely that you will be able to downregulate and distance yourself easier from experienced strong feelings and ruminative thoughts after medication intake’. In order to assess to which extent observed effects in the treatment groups are due to a placebo response, a no treatment control group (CG) was realized.

### 2.3 Experimental procedure

At the beginning of the procedure, participants were informed about the study according to a cover story, suggesting that the study is about the examination of affect regulatory effects of a supposedly antidepressant nasal spray called ‘Doluxefin-direkt’ in an emotion inducing paradigm. Participants were informed that they would be assigned to one of three possible groups: the antidepressant nasal spray, a placebo group or control group when in fact they were assigned to one of the three conditions mentioned above. Subsequently, participants received detailed written descriptions of the medication’s mechanisms mode of action. After giving written informed consent, the participants were informed about the overall procedure of the study. Subsequently, the blood pressure of each participant was measured as part of the cover story. Before the process of randomization, participants filled out different questionnaires regarding their current affective state and tendency to ruminate, levels of depressive symptoms and shame proneness. Then, all participants were randomly assigned to one of the three experimental groups, by choosing a random envelope within a box, containing a small piece of paper where their group assignment was written. Due to study design, investigators and participants of the control group were not blinded and only participants of the deceptive-placebo groups were masked. Participants of the placebo conditions received a treatment (DP-A or DP-R) with specific verbal instructions from study investigators (see 2.2.) and participants of the control group received no treatment (CG) and continued directly with the study procedure. Subsequently, shame was induced in participants through an autobiographic recall adapted from De Hooge et al. (2008). Participants were asked to recall and write in detail about a past event at which they felt particularly inadequate, ashamed, or inept for about 10 min. Afterwards current experience of shame, rumination and cognitive flexibility were assessed again. At the end of the study investigators conducted a follow-up interview to assess treatment credibility and experienced side effects. Afterwards, participants were told about the cover story and deception and then informed about the actual aims of our study. Each experimental investigation was conducted by one of the three involved psychology master students at a laboratory room at the Psychology Department of the University of Marburg.

**Figure 1:**
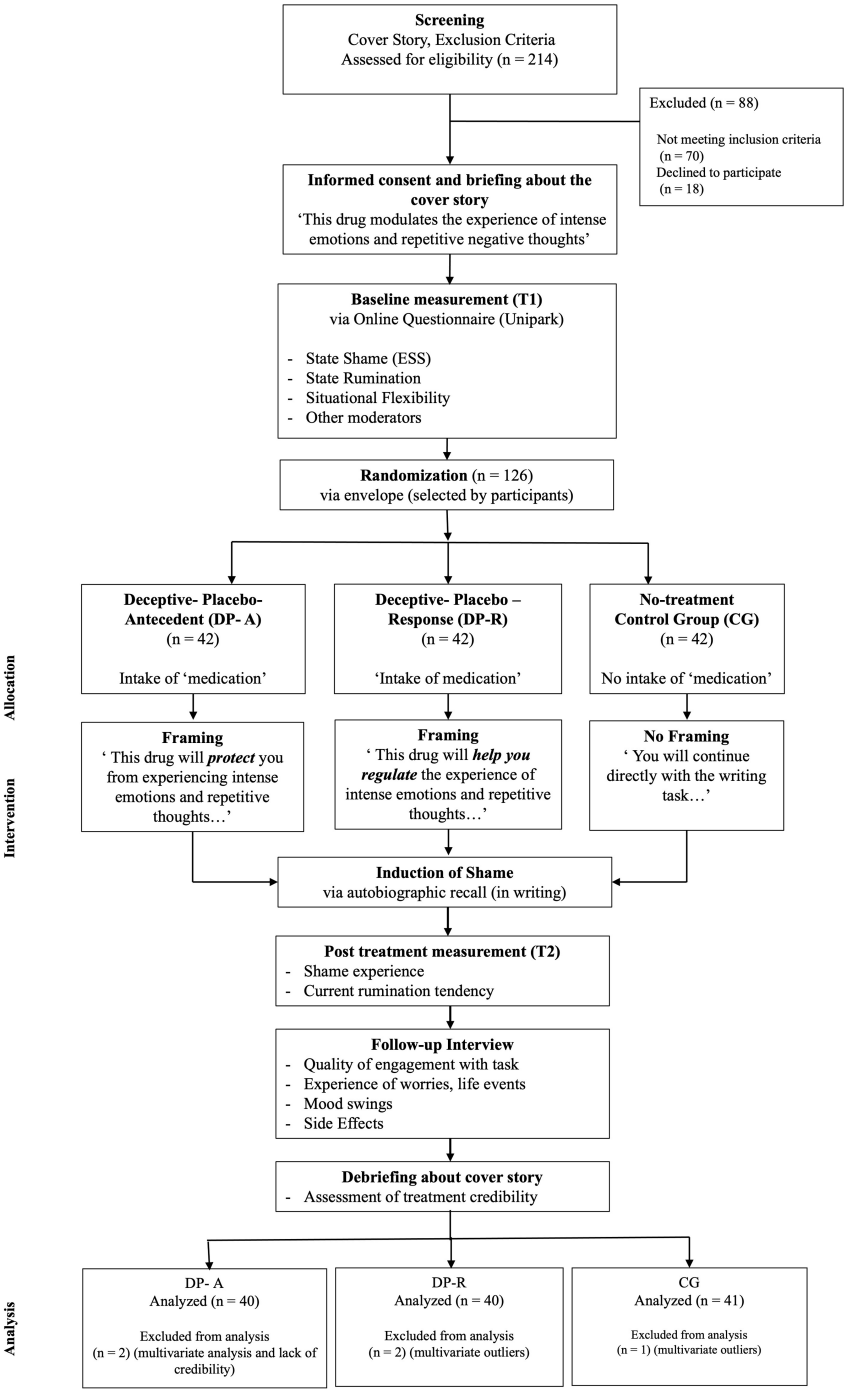
Illustration of the study design. All participants passed through a baseline assessment were afterwards assigned randomly to one to three groups: one group received a deceptive placebo with an antecedent-focused regulation instruction (DP-A), one group received a deceptive placebo with a response-modulating regulation instruction (DP-R) whereas the third group received no treatment (control group CG). Consequently, shame was induced, and current shame experience, rumination and situational flexibility were measured again. Afterwards participants answered questions in a follow-up interview. At the end of the experiment participants were debriefed about the cover story and actual study goals.

For an overview see Figure 1.

### 2.4 Measures

Questionnaires were completed on the computer via the survey software Unipark.^1^

#### 2.4.1 State shame

State shame was assessed using the German version of Turner’s Experiential Shame Scale (ESS, Turner, 2014; German version: Rüsch and Brück, unpublished), an 11-item self-report questionnaire that measures physical, emotional, and social markers of a momentary shame-reaction. Items are rated from 1 to 7 (e.g., “Physically, I feel … pale/flushed,” “Emotionally, I feel … content/distressed,” “Socially, I feel like being … sociable/hiding”). The ESS demonstrated satisfactory internal consistency of 0.74–0.81. The internal consistency in this sample was *α* = 0.72–0.79 across administrations.

#### 2.4.2 Current rumination and cognitive flexibility

To measure the current rumination tendency as well as cognitive flexibility of participants during the study a German questionnaire (‘Fragebogen zur Erfassung aktueller Ruminationsneigung’) by de Jong-Meyer et al. (2009) was used.

This questionnaire consists of 8 items, of which 4 items load on two different factors each for rumination (called ‘current rumination’) and cognitive flexibility (called ‘situational flexibility’). Both factors are significantly negatively correlated with each other (*r* = −0.48, *p* < 0.01). Items are rated on a10-point Likert scale from 0 (‘It does not at all apply’) to 10 (‘It does apply really well’). Both scales demonstrated acceptable reliability with an internal consistency of *α* = 0.79–0.87. In the present study internal consistency for ‘current rumination’ is *α* = 0.83 at T1 and *α* = 0.88 at T2 and for ‘situational flexibility’ *α* is 0.85 at T1 and 0.87 at T2.

#### 2.4.3 Shame proneness

The German version of the Personal Feelings Questionnaire-2 (PFQ-2, Harder and Zalma, 1990; German version from Rüsch and Brück, unpublished) was used to assess shame proneness. The PFQ-2 is a 22-item self-report measure using a 5-point response format ranging from 0 to 4 asking about the frequency of experienced feelings. The questionnaire is designed to assess shame- (10 items) and guilt- proneness (6 items). The shame scale of the PFQ-2 showed satisfactory internal consistency (*α* = 0.78). Cronbach’s alphas for the shame scale in the present study is *α* = 0.82.

#### 2.4.4 Depressive symptoms

The distribution of depressive symptoms was assessed with the Patient Health Questionnaire module for depression (PHQ-9) by Kroenke et al. (2002). It was developed as a brief screening instrument for depressive symptoms using 9 items which are rated on a 4-point Likert scale. Internal consistency for PHQ-9 scale in the present study is *α* = 0.79.

#### 2.4.5 Emotional control

The 32 items of the German adaptation Emotional Control Questionnaire 2 [ECQ2-D, Roger and Najarian, 1989; German version by Tausch (1996)] assess individual differences in response to emotional arousal on 4 scales: rehearsal, emotional inhibition, impulsivity and anger control. Emotion control was also rated on a 4-point frequency scale (1–4). The present study used the sum score to indicate the extent of emotional control. The internal consistency for the ECQ2-D sum score in the present study is *α* = 0.78.

#### 2.4.6 Experiential avoidance

Experiential avoidance was assessed using the German adaptation of the Acceptance and Action Questionnaire (AAQ, Hayes et al., 2004; German version by Rüsch and Brück, unpublished). This 9-item questionnaire measures tendencies for negative evaluations, avoidance, the need, and desire to control or the inability to take action in the face of negatively evaluated private events using a 7-point Likert scale. The scale shows good reliability and validity in clinical and non-clinical samples (e.g., Hayes et al., 2004). Internal consistency in the present study is *α* = 0.74.

#### 2.4.7 Side effects

At the follow-up interview the experience of side effects was assessed. Participants were asked to report whether they experienced any form of irritations in nose or throat, tiredness, nausea, coldness in hands or feet, headache, or other side effects.

#### 2.4.8 Credibility

Every participant was asked to report any impressions or notes to the overall study procedure or medication intake (if applicable) in order to assess treatment credibility. Afterwards, the treatment credibility was rated by study investigators in the categories ‘believed it definitely’, ‘rather believed it’ or ‘did not believe it at all’.

### 2.5 Statistical analysis

There were no missing values due to the study design which allowed participants only to continue if they entered all values. Statistical outliers regarding primary outcomes on state rumination, cognitive flexibility and shame scores were identified by standardized z-values and their histograms for univariate outliers or via Mahalanobis distance for multivariate outliers (Eid et al., 2010).

To investigate whether both groups differed in baseline values of primary outcomes a multivariate analysis of variance (MANOVA) was conducted with ‘condition’ as the independent variable (IV) and baseline values of state shame, state rumination and cognitive flexibility as well as values of age, shame proneness, emotional control, experiential avoidance and depressive symptoms as dependent variables (DVs). Further the distribution of gender, educational level and employment status between experimental groups was examined via chi-square tests.

For analyses of changes in outcomes of state shame, state rumination and cognitive flexibility separate mixed-effect ANOVAs were performed with ‘timepoint’ and ‘condition’ as IVs. In case of significant results, post-hoc analysis was performed using planned contrast coding in order to assess the extent to which the experimental groups differed from one another in primary outcomes. For the first contrast both treatment groups (DP-A and DP-R) were compared to the no-treatment control group (−1, −1, 2) and for the second contrast both placebo groups were compared with each other (−1, 1, 0). In order to avoid alpha-error inflation Bonferroni-Holm corrected *p*-values were reported.

To assess possible modulatory influences, measures of depression, shame proneness, emotional control and experiential avoidance were added as centered predictors in the ANOVAs, respectively. Analyses for each outcome with respective modulatory influences will be reported separately.

All analyses were performed using R 4.2.1 and R Studio 2022.07.2.

## 3 Results

### 3.1 Sample characteristics

For the study a total of 126 participants were recruited. All participants fulfilled the inclusion criteria for this study. Four of them were identified as multivariate outliers on primary outcomes and were excluded from further analyses. One participant had to be excluded due to a reported incredibility of the experiment. A sensitivity analysis including outliers was performed. The results of the analysis indicated no changes in reported result pattern. All experimental groups were of similar size, that is 40 participants in the treatment groups and 41 participants in the no-treatment control group. Overall, the final sample size consisted of 71 women (58.68%), 48 men (39.67%) and 2 diverse (1.65%).

On average, participants were 24.12 years old (SD = 6.99) ranging from 18 to 75 years. With regard to the educational level, the sample consisted mainly of students (89%).

Although scores ranged from 0 to 24, participants reported only minimal depressive symptoms on the PHQ-9 Sum Score of (*M* = 7.0, SD = 4.3) according to Kroenke et al. (2002). Further, participants exhibited an inconspicuous manifestation of shame proneness (*M* = 12.7, SD = 6.1) according to Rüsch et al. (2007) and of experiential avoidance (*M* = 33.0, SD = 8.6) according to Hoyer and Gloster (2013).

Table 1 presents demographic sample characteristics.

**Table 1:** Descriptive statistics of sample characteristics and baseline values.

Variables	DP-A (*N* = 40)	DP-R (*N* = 40)	CG (*N* = 41)
Age, *M* (SD)	23.88 (4.77)	25.13 (10.37)	23.39 (4.22)
PHQ sum score, *M* (SD)	7.28 (4.21)	6.90 (4.45)	6.83 (4.40)
PFQ sum score, *M* (SD)	14.10 (6.81)	11.83 (4.80)	12.95 (5.95)
AAQ sum score, *M* (SD)	33.98 (8.72)	31.50 (8.46)	33.49 (8.54)
ECQ2-D sum score, *M* (SD)	85.65 (8.26)	88.28 (8.22)	87.61 (8.30)
Sex, *N* (%)			
Male	16 (40)	16 (40)	16 (39.02)
Female	24 (60)	23 (57.50)	24 (58.54)
Diverse	0 (0)	1 (2.50)	1 (2.44)
Educational level, *N* (%)			
Secondary education	1 (2.50)	2 (5)	3 (7.32)
High school degree	30 (75)	27 (67.50)	31 (75.61)
University degree	9 (22.50)	11 (27.50)	7 (17.07)
Profession, *N* (%)			
Student	37 (92.5)	35 (87.5)	36 (87.80)
Employee	3 (7.5)	5 (12.5)	5 (12.20)
State shame sum score T1, *M* (SD)	34.43 (7.80)	32.38 (7.15)	33.66 (7.24)
Current rumination score T1, *M* (SD)	16.08 (9.17)	15.75 (8.92)	16.46 (6.96)
Situational flexibility score T1, *M* (SD)	29.18 (7.27)	30.15 (8.42)	30.32 (6.24)

M, Mean; SD, Standard deviation; N, Number of participants; %, Percentage of participants; DP-A, Deceptive Placebo – antecedent is a placebo treatment with an antecedent-focused instruction of affect regulation; DP-R, Deceptive Placebo – Response is a placebo treatment with a response-modulating instruction of affect regulation; CG, control group without treatment.

### 3.2 Examination of baseline differences

Regarding the baseline measurements no significant differences between the experimental groups were observed (MANOVA *F*(2,118) = 0.660, *p* = 0.848, *ɳ^2^_p_* = 0.05). The distribution of gender was not significantly different across the three groups (*χ^2^*(4) = 0.1.016, *p* = 0.907, *φ* = 0.068), nor was the distribution of educational level (*χ^2^*(6) = 2.665, *p* = 0.850, *φ* = 0.10), and employment status (*χ^2^*(*2*) = 0.281, *p* = 0.869, *φ* = 0.033).

### 3.3 Main findings

#### 3.3.1 State shame

Results of the mixed-effects ANOVA indicated that the reported significant increase in shame after the autobiographic recall (Time: *F*(1,118) = 5.059, *p* = 0.026, *ω^2^* = 0.03) differed between the experimental groups (Time × Group: *F*(2,118) = 3.696, *p* = 0.034, *ω^2^* = 0.03).

**Figure 2:**
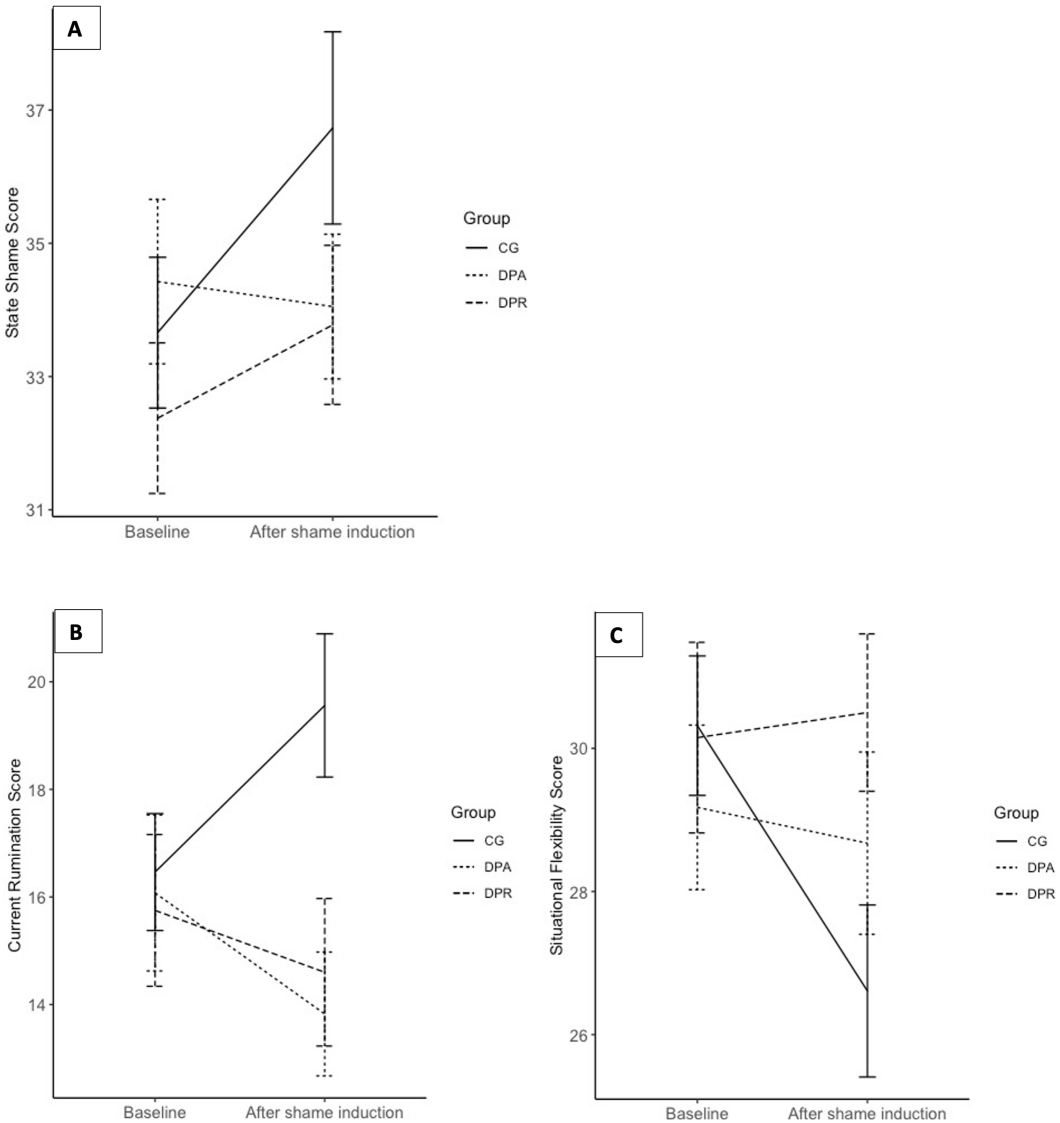
**(A)** Comparison of state shame scores over time across experimental groups. Time points are indicated on the x-axis and averaged sum scores of current rumination are indicated on the y-axis. Scores can range from 10 to 70. Error bars show the standard error of the mean (SEM). **(B)** Comparison of current rumination scores over time across experimental groups. Time points are indicated on the x-axis and averaged sum scores of current rumination are indicated on the y-axis. Scores can range from 10 to 40. Error bars show the standard error of the mean (SEM). **(C)** Comparison of situational flexibility scores over time across experimental groups. Time points are indicated on the x-axis and averaged sum scores of situational flexibility are indicated on the y-axis. Scores can range from 10 to 40. Error bars show the standard error of the mean (SEM). DP-A (Deceptive Placebo- Antecedent) and DP-R (Deceptive Placebo Response) group differed in their instruction of efficacy: DP-A Placebo group was told that the 'drug' would protect against experiencing intensive feelings and ruminative thoughts. DP-R Placebo group was told that the 'drug' would help to regulate the experience of intensive feelings and ruminative thoughts. CG was a no-treatment control group. Shame was induced via an autobiographical recall.

Subsequent analysis of Bonferroni-Holm corrected planned contrasts revealed that participants of the control group reported increased shame levels after intervention in comparison to participants of both DP-A and DP-R group (*b* = 3.08, SE = 0.81, 95% CI = [0.80–5.92], *p* = 0.044, *ω^2^* = 0.03). The changes in the DP-A group did not differ significantly from the DP-R group (*b* = −1.78, SE = 1.49, 95% CI = [−5.69–2.14], *p* = 0.699, *ω^2^* < 0.02). For further detail see Figure 2.

Analyses of modulatory influences indicated a significant effect of the covariate ‘experiential avoidance’ on treatment effects regarding shame experience (*F*(2,115) = 3.470, *p* = 0.031). Further inspection of the mixed effects model revealed significant stronger treatment effects for the antecedent-oriented regulation strategy in the reduction of shame reports within participants with higher experiential avoidance in comparison to the control group (*t*(115) = −2.478, *b* = −0.42, 95% CI = [−0.75 – −0.09], *p* = 0.014). However, no significant modulatory differences of experiential avoidance on changes in state shame scores between both placebo groups could be found (*t*(115) = −1.209, *b* = −0.21, 95% CI = [−0.54–0.13], *p* = 0.228).

No overall modulatory influences of levels of shame proneness (*F*(2,115) = 2.269, *p* = 0.764), depression (*F*(2,115) = 2.504, *p* = 0.086) and emotional control (*F*(2,115) = 1.073, *p* = 0.345) on the outcome of state shame could be observed.

#### 3.3.2 Current rumination

Mixed-effects ANOVA analyses indicated that as expected, participants ruminated more after the autobiographic recall and that changes in reports of current rumination were significantly different between experimental groups (Time × Group: *F*(2,118) = 7.581, *p* < 0.001, *ω^2^* = 0.10). Planned contrasts of mixed-effects ANOVA analyses indicated that participants of the control group reported significantly more rumination after intervention in comparison to both placebo groups (*b* = 4.80, SE = 1.26, 95% CI = [1.50–8.09], *p* ≤ 0.001). No significant differences between the DP-A group and the DP-R group in state rumination score changes could be observed (*b* = −1.10, SE = 1.46, 95% CI = [−3.98 – 1.78], *p* = 0.453). For more detailed illustration of results see Figure 2.

Further analyses revealed influences of the covariate ‘emotional control’ on treatment effects of the outcome current rumination *F*(2,115) = 4.588, *p* = 0.012). Participants who reported higher levels of emotional control ruminated more in the D-PA group after autobiographical recall when compared with participants of the other placebo condition DP-R (*t*(115) = 2.017, *p* = 0.046) and the no-treatment CG as well (*t*(115) = 2.968, *p* = 0.004). No modulatory influences of levels of shame proneness (*F*(2,115) = 1.555, *p* = 0.216) and depression (*F*(2,115) = 2.668, *p* = 0.074) as well as experiential avoidance [(*F*(2,115) = 1.415, *p* = 0.247] were found.

#### 3.3.3 Cognitive flexibility

As indicated by the results of the mixed-effects ANOVA participants reported less cognitive flexibility after the autobiographic recall (Time: *F*(1,118) = 5.215, *p* = 0.024, *ω^2^* = 0.03). Again, group differences for changes in cognitive flexibility could be observed (Time × Group: *F*(2,118) = 4.851, *p* = 0.009, *ω^2^* = 0.06). Subsequent inspection of planned contrasts revealed that participants of the CG reported less cognitive flexibility after shame induction in comparison to both DP-A and DP-R group (*b* = −3.63, SE = 1.19, 95% CI = [−6.75 – −0.51], *p* = 0.011). Again, both deceptive placebo conditions did not differ significantly from each other in pre to post intervention changes (*b* = −0.85, SE = 1.38, 95% CI = [−3.58–1.88], *p* = 0.540). For further detail see Figure 2.

No modulatory influences of symptoms of depression and personality traits on cognitive flexibility could be observed.

#### 3.3.4 Follow-up interview: prior experience, engagement, and credibility

Across all groups no issues with task engagement could be registered. The majority of participants of the placebo treatment groups reported to have experienced bodily side effects, predominantly a light irritation of nose and throat. When asked about credibility of the cover story, meaning the experimental setup and supposed treatment, 109 of 121 participants (91%) reported to have definitely believed it and the remaining 9 participants reported to have rather believed it. Most participants (69.4%) reported no negative life events (e.g., loss of a closely related person, a straining break up or similar distressing events) within the last month. Only 15.7% of participants expressed the experience of worries after the autobiographic recall. However, participants of the control group reported twice as many experienced worries after the intervention (24.4%) as compared to the placebo groups.

The descriptive results of the Follow-Up interview are displayed in Table 2.

**Table 2:** Descriptive statistics of follow-up interview outcomes.

Variables	All (*N* = 121)	DP-A (*N* = 40)	DP-R (*N* = 40)	CG (*N* = 41)
Currently negative life event, *N* (%)	37 (30.6)	13 (32.5)	11 (27.5)	13 (31.7)
Engagement with task, *N* (%)	121 (100)	40 (100)	40 (100)	41 (100)
Experienced worries during task, *N* (%)	19 (15.7)	4 (10)	5 (12.5)	10 (24.4)
Experienced side effects, *N* (%)				
Light irritation of nose and throat	55 (45.5)	27 (67.5)	28 (70)	-
Fatigue	2 (1.7)	1 (2.5)	1 (2.5)	
Nausea	1 (1)	1 (2.5)	0 (0)	
Cold sensations of hands & feet	1 (1)	1 (2.5)	0 (0)	
Headache	2 (1)	2 (5)	0 (0)	
Other	10 (8.3)	5 (12.5)	5 (12.5)	

N, Number of participants; %, Percentage of participants; DP-A, Deceptive Placebo – antecedent is a placebo treatment with an antecedent-focused instruction of affect regulation; DP-R, Deceptive Placebo – Response is a placebo treatment with a response-modulating instruction of affect regulation; CG, control group without treatment.

## 4 Discussion

### 4.1 Summary and discussion of results

Consistent with our hypotheses a deceptive placebo response could be observed on shame as well as rumination and cognitive flexibility after the intervention when compared to a no treatment control group. No differences between antecedent and response-oriented framing in the deceptive placebo response could be observed. Experiential avoidance modulated the DP-A placebo response on shame experience and emotional control modulated the DP-A placebo response on rumination processes. No modulatory effects of depression and shame proneness were found. In line with our previous study of Rebstock et al. (2020) the effects of deceptive placebo administration on a negative affect and rumination could be replicated. Moreover, the results of the present study indicate a placebo response on reports of cognitive flexibility as well. Similarly, participants with placebo treatment reported treatment credibility as well as side effects such as bodily sensations in the follow-up interview which again suggests a successful experimental manipulation of expectations through deceptive placebo treatment in the present study. However, in contrast to the Rebstock et al. (2020) study, the present study focuses on comparing two different placebo framings on the affect shame and rumination using another experimental induction method than in the previous study. Results indicate that the detailed autobiographical recall of past experiences associated with feelings of shame and insufficiency may not only induce shame but also rumination processes as suggested by previous research (Watkins and Roberts, 2020).

In contrast to our hypothesis, however, no significant difference between the two placebo conditions (antecedent vs. response orientation) could be found. These findings suggest that placebo-induced expectancy to receive a potent and affect-regulatory medication is sufficient to decrease experiences of aversive affect or symptoms independent from the instructed mode of action (antecedent regulation vs. response regulation). Yet, our data indicate that the short time experimental mood manipulation did not elicit strong feelings of shame, therefore potentially concealing a modulating effect of the different regulation strategies. It seems conceivable, that participants of both groups were well aware of the temporary and artificial aspect of the experimentally induced shame. Several studies demonstrated the short-term effect of experimental mood inductions (Westermann et al., 1996).

However, our findings are still in line with current research suggesting that deceptive placebo regulate aversive affect and cognitive processes such as rumination and provide further evidence of expectancy mechanisms underlying the placebo response (Petrie and Rief, 2019; Rebstock et al., 2020; Geers et al., 2021).

In accordance with previous research our findings on experiential avoidance suggest a positive association between experiences of shame with avoidance tendencies (Tangney, 1991; Tangney et al., 1996; Budiarto and Helmi, 2021). However, no significant difference in the post-hoc analysis of DP-A response against the DP-R response could be observed, so this result should be interpreted with caution.

Further, our findings on modulatory influences of emotional control on the antecedent-focused placebo treatment in the context of rumination are in accordance with rumination research that suggests that rumination processes themselves are a dysfunctional approach to avoid aversive situations and the responsibility to take action (Nolen-Hoeksema et al., 2008).

Our findings on symptoms of depression or shame proneness could be explained by specific sample characteristics in the present study. In fact, our rather young and healthy study sample exhibited average levels of shame proneness and depression which possibly are too low to produce modulatory influences in treatment efficacy of shame, rumination and cognitive flexibility.

### 4.2 Limitations

The generalizability of our findings to the efficacy of deceptive placebo treatment on affect regulation through manipulation of expectations may be limited by several features of this study.

Our study procedure assessed state shame by self-report without objective measures of skin conductance or heart rate variability etc. Thus, our findings on deceptive placebo effects only apply to subjective reports of shame experience and no conclusions on potential placebo responses in the context of bodily reactions typically associated with shame (sweating, blushing, higher heart rate levels etc.) can be drawn.

In our study design we compared both experimental placebo groups to a no treatment control group and not an additional placebo control condition without instruction. Thus, our findings on the extent of additional effects of investigated instructions on the placebo response are limited. Participants of the placebo groups received specific instructions regarding treatment efficacy as the ‘medication’ was administered openly which could have activated conditioning processes which are considered as one of the main psychological mechanisms for inducing a placebo response (Petrie and Rief, 2019). No additional control group receiving an open administered placebo (OLP) was created in the present study. Therefore, conditioning effects on reported placebo responses cannot be ruled out.

Although we consider depression as a continuous variable, our sample does not cover the full range of this variable and cannot be interpreted in a clinical meaning. Therefore, results on depression as a moderator should be interpreted with caution.

Because the placebo instructions were given by study investigators, an investigator bias such as the Rosenthal effect cannot be ruled out (Rosenthal, 1964). Experimental manipulation was based on the delivery of specific instructions as well, therefore it is possible that variance in performance could have also influenced results. However, particular care was taken by all study investigators to deliver instructions in a standardized manner using prior developed manuals for each experimental group.

Further, the effects were investigated in a healthy study sample possibly reducing the power to find a small effect. Thus, these findings should be interpreted with caution.

Generalizability of this study is further restricted due to the highly educated and healthy study sample which is not representative of the general. Thereby, transferability of results onto clinically individuals is in question and should be investigated in future studies.

### 4.3 Future perspectives

A basic understanding of mechanisms influencing the placebo response is essential for optimizing treatment of various clinical disorders and symptoms. The present study is the first, to our knowledge, to have adopted and developed an experimental paradigm for investigating and comparing two affect-regulatory characteristics of placebo-induced expectancy effects in the context of shame, rumination and cognitive flexibility under highly controlled laboratory conditions. Effects of expectancy induction were demonstrated in reports of shame, current rumination tendency and cognitive flexibility after shame was induced. However, differences between placebo treatments could only be observed partially in the context of modulatory influences of personality traits.

As reports of the follow-up interview suggest the experience of aversive side effects (headache, level-headedness etc.), our study design could be refined by creating an additional control group, varying the mode of instruction (positive, neutral, negative) and investigating the nocebo effect. Effects of affect regulatory strategies in the context of nocebo responses have not yet been subject to empirical investigations.

Due to the experimental design, the present study only provides evidence for short-time efficacy of the used placebo treatments on experimentally induced changes in shame and cognitive states. Future studies should test possible long-term effects of the affect-regulatory placebo interventions and if possible, include the assessment of other negative affect or pain as these are closely linked to emotion regulation strategies (Totterdell and Holman, 2003; see review Koechlin et al., 2018) and placebo responses as suggested by current research (Klinger et al., 2014; Geers et al., 2021).

## 5 Conclusion

Our hypotheses regarding the efficacy of deceptive placebo treatment on shame experience as well as rumination and cognitive flexibility could be confirmed. Modulatory influences of experiential avoidance and emotional control indicate different processes in efficacy of both placebo treatments in the context of shame experience and rumination.

The present study provided further evidence for a deceptive placebo response in the context of shame, current rumination and cognitive flexibility.

The findings of the present study are of relevance to the utilization of placebo effects in clinical practice to enhance treatment outcomes as they demonstrate great efficacy for various clinical symptoms. Framing of treatment instructions, for example antecedent-oriented regulation vs. response-modulating orientation within the practitioner-patient communication should be considered, especially when dealing with psychological comorbidities and certain personality traits. The use of placebo induced expectancies could stabilize or even increase treatment adherence.

Finally, further research investigating framing effects of placebo treatment is needed, particularly under clinical conditions and with respect to individual personality traits and coping styles.

## Data Availability

The raw data supporting the conclusions of this article will be made available by the authors, without undue reservation.

## Footnotes

^1^ www.unipark.com
